# The Parathyroid Hormone-Related Protein/Parathyroid Hormone 1 Receptor Axis in Adipose Tissue

**DOI:** 10.3390/biom11111570

**Published:** 2021-10-22

**Authors:** Adriana Izquierdo-Lahuerta

**Affiliations:** Area of Biochemistry and Molecular Biology, Department of Basic Sciences of Health, Faculty of Sciences of Health, Campus of Alcorcón, University Rey Juan Carlos, 28922 Madrid, Spain; adriana.izquierdo@urjc.es

**Keywords:** PTHrP, PTH1R, PPARgamma, adipose tissue

## Abstract

Adipose tissue is an organ that shows great plasticity and is able to adapt to the conditions to which the body is subdued. It participates in the regulation of energetic homeostasis and has endocrine functions. Recent studies have shown how the parathyroid hormone-related protein (PTHrP)/Parathyroid Hormone Receptor 1 (PTH1R) axis participates in the regulation of adipogenesis, opposing the action of Peroxisome proliferator-activated receptor gamma (PPARγ). In addition to this, PTHrP is overexpressed in adipose tissue in situations of wear and tear of the body, favoring browning and lipolysis in this tissue. It is also overexpressed in adipose tissue in stressful situations but in the opposite direction, in obesity, metabolic syndrome, type 2 diabetes mellitus (T2DM) and gestational diabetes mellitus (GDM). In conclusion, the PTHrP/PTH1R axis has a main role in adipose tissue, participating in its differentiation and remodeling. PTHrP might be used in obesity treatment and its complications for its ability to reprogram adipogenesis and adipose tissue expansion, WAT browning and for the improvement of the insulin sensitivity. In addition, PTHrP could even be used as a marker of placental status and maternal adaptations to prevent future metabolic problems in mothers and children, as well as in the treatment of bone-related diseases such as osteoporosis.

## 1. Introduction

Parathyroid hormone-related protein (PTHrP) was discovered in 1987 as a factor humoral hypercalcemia of malignancy (HHM). PTHrP is encoded by the *PTHLH* gene, located in humans on chromosome 12 (12p11.22), and is expressed in diverse tissues, whereas the gene encoding parathyroid hormone (PTH) is located on chromosome 11, is only transcripted in the parathyroid glands and regulates calcium homeostasis in the body. The human PTHrP gene produces three isoforms of 139, 141 and 173 residues by alternate splicing. The translation and processing produce three matures peptides, the N-terminal, mid-region and C-terminal mature peptides, as well as combinations of these forms, each having distinct autocrine and paracrine bioactivities [1]. The N-terminal domains of PTH and PTHrP are very similar; eight of the first 13 amino acid residues are identical and promote similar effects. These effects are produced by the binding of the N-terminal domain of both peptides with their common Parathyroid Hormone 1 Receptor (PTH1R). PTH1R is a class II G-protein coupled receptor with seven transmembrane domains. In addition to the PTH1R, a second receptor has been identified, the PTH2R [2], and this receptor interacts with PTH and with a hypothalamic peptide called the tuberoinfundibular peptide of 39 residues (TIP 39), the third member of the single gene family [3]. Thus, PTHrP binds and activates PTHR1 but not PTHR2. PTH can bind and activate both PTHR1 and PTHR2. TIP39 can bind to and activate PTHR2, but not PTHR1 [1,2].

PTHrP is expressed in diverse tissues and regulates calcium transport in the kidney, bone and placenta; it regulates a smooth muscle tone and regulates cell proliferation, cell differentiation and cell death [1]. PTH1R is widely expressed in the bones and kidney, where it mediates the classical effects of PTH and PTHrP on calcium homeostasis. PTH1R is also expressed at low levels in other tissues, where it acts autocrine or paracrine in response to local PTHrP production. The presence of PTHrP and PTH1R genes in the common ancestor of the chordates and the lethality of both knockout mice highlights the crucial roles of the axis PTHrP/PTH1R during development as an adult physiological function.

In the last decades, it has been seen that adipose tissue is not a simple stocker of fat, demonstrating its influence on the immune system and other additional functions. Today, adipose tissue is considered an endocrine organ since it secretes numerous bioactive molecules called adipokines, such as adiponectin and leptin, into the blood circulation. Adipose tissue is considered a dynamic tissue, able to adapt to different environmental, nutritional, and energy needs of the organism [4]. Its function is to be responsible for energy storage, nutrient detection, and body temperature regulation. There are mainly two types of adipose tissue, white adipose tissue (WAT) and brown adipose tissue (BAT). WAT is highly vascularised and innervated and plays a key homeostatic role, not only by ensuring an efficient energy storage but also by its quick mobilisation (lipids) to ensure peripheral demands [5]. WAT occurs in localised depots in two compartments of the body: some are below the skin (subcutaneous WAT) and in the trunk (visceral WAT) [4]. BAT is differentiated at the morphological/molecular level and by its unique thermogenic capacity [5].

In this review, our aim is to show the role of the PTHrP/PTH1R axis in adipose tissue, not only in the adipocyte formation (adipogenesis) but also in its adaptations both to obesity and to diseases that cause the wear and tear of the organism, such as cancer or chronic kidney disease (CKD).

## 2. PTHrP/PTH1R in Adipogenesis vs. Osteogenesis

White and brown adipogenesis require a complex coordination of multiple regulatory and signaling pathways. Adipocytes and osteoblasts originate from the same bone marrow stromal pluripotent stem cells. Depending on a balance through the regulating key transcription factors to which these cells are subjected, they will follow one or another path of differentiation [6]. 

PTH and PTHrP have been shown to have an anabolic role in bones. These factors are capable of promoting the proliferation of osteogenic progenitor cells, of enhancing osteoblast differentiation, and of inhibiting apoptosis of osteoblastic cells, while blocking adipogenesis by the repression, among others, of peroxisome proliferator-activated receptor gamma (PPARγ) [7,8]. In this sense, heterozygous mice PTHrP-null alleles show a premature osteoporosis associated with an increased bone marrow adiposity [9]. These anabolic effects occur at the stages of osteogenesis and adipogenesis, respectively [10]. Recent gene deletion studies have shown that osteoblast differentiation requires a complex sequence of processes to activate transcription factors involved in osteoblastogenesis, including Wnt/β-catenin, Runt-related transcription factor 2 (Runx2), and Osterix, and to suppress transcription factors involved in adipogenesis (including PPARγ and CCAAT/enhancer binding protein (C/EBP)) [11].

**Figure 1:**
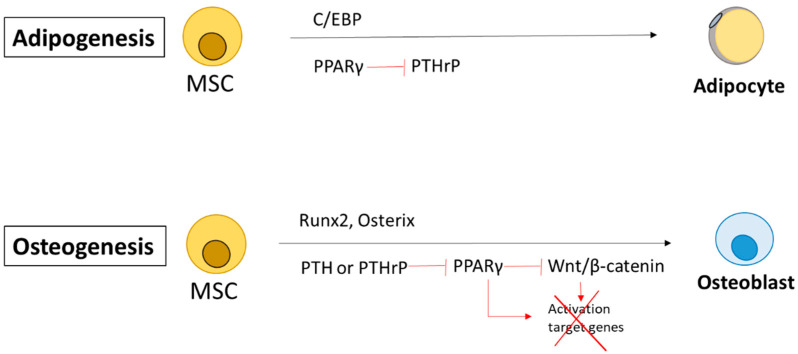
Reciprocal relation between PTHrP and PPARgamma in adipogenesis and osteogenesis. Adipogenesis requires activation of C/EBP and PPARγ, and PPAR binds to *Pthlh* promoter regions as a repressor. Osteogenesis requires activation of transcription factors such as Wnt/β-catenin, Runx2, and Osterix. C/EBP: CCAAT/enhancer binding protein MSCs: mesenchymal stem cells; PTHrP: Parathyroid hormone-related protein; RunX2: Runt-related transcription factor 2.

PPARγ, a master controller of the adipogenic program, including adiposity differentiation, triglyceride storage, energy homeostasis and insulin sensitivity [12], seems to have contrary effects in the process of differentiation of mesenchymal stem cells (MSC). In humans, the PPARγ gene encodes two protein isoforms (γ1 and γ2). PPARγ1 is expressed in many tissues and cell types, including white and brown adipose tissue, skeletal muscle, liver, pancreatic cells, macrophages, colon, bone and placenta. By contrast, PPARγ2 has a restricted expression in white and brown adipose tissue under physiological conditions, although it is induced in other tissues in response to overnutrition or genetic obesity [12,13]. PPARγ is required for the preadipocyte differentiation of adipose tissue, the control of the adipocyte number, and their activation, which stimulates the storage of fatty acids in mature adipocytes and other tissues as well [13]. PPARγ induces adipocyte differentiation in fibroblasts and myoblasts as well as in bone marrow stromal cells. By contrast, PTHrP protein expression is detected in undifferentiated mesenchymal cells and decreases progressively during adipogenesis. It seems that there is a reciprocal control relationship between PTHrP and PPARγ (Figure 1). On the one hand, it has been described how the activation of PTHrP is capable of diminishing the capacity of PPARγ to act over their target genes, not affecting the transcription of PPARγ [7]. On the other hand, it has recently been described how PPARγ binds to Pthlh promoter regions as a repressor in immortalized mouse lung epithelial cells. The Pthlh gene is significantly increased during E14.5 to E18.5 in lung-specific Pparg conditional knockout mice [14]. Furthermore, in the lung, alveolar epithelial cells produce PTHrP. This signal is picked up by the PTH1R receptor expressed on lipofibroblasts (LIF), inducing the expression of adipose differentiation-related protein (Adrp) via the PPARγ pathway and promoting the secretion of triglycerides and leptin essential for surfactant production [15].

In addition, diet seems to also influence the expression of the factors to which the cells are subjected when “deciding” between adipogenesis or osteogenesis. In this sense, it has been shown in MC3T3-E1 and HEK293 cells that long chain polyunsaturated fatty acids (LCPUFAs) are also able to activate the PTH1R receptor and act synergistically with PTH(1–34), promoting osteogenesis [16]. This action of PTH/PTH1R could be used as some sort of treatment for bone diseases such as osteoporosis.

## 3. PTHrP/PTH1R and Adipose Tissue

Adipose tissue is a dynamic organ, responsible for energy storage, nutrient sensing, and regulating body temperature. Fundamentally, there are two types of adipose tissue, with different functions: (1) the white adipose tissue (WAT) located in several depots, which stores lipids in the form of triglycerides and participates in isolation and mechanical protection; (2) the brown adipose tissue (BAT), whose function is to dissipate energy in the form of heat (thermogenesis) [4]. In recent years, it has been seen that adipose tissue is not a mere deposit of fat; it also acts as an endocrine tissue capable of secreting cytokines termed as adipokines (such as leptin, adiponentin, resistin, Retinol-binding protein 4 (RBP4), omentin and nesfatin) and lipid metabolites, called lipokines (such as C16:1n7-palmitoleate) [4,5,17]. In this way, the adipose tissue shows great plasticity and dynamic regulation, adapting to the energetic conditions of the organism. When there is an energetic demand, it mobilises lipids from adipocytes, activating lipolysis and fatty acid β-oxidation, and releasing fatty acids and glycerol into the bloodstream. On the contrary, when there is an excessive energy supply and the expenditure decreases, the excess energy accumulates in the form of triglycerides. This plasticity and endocrine function of adipose tissue explains its ability to remodel from white to brown (WAT browning) and vice versa (BAT whitening) [4,5,17]. 

**Figure 2:**
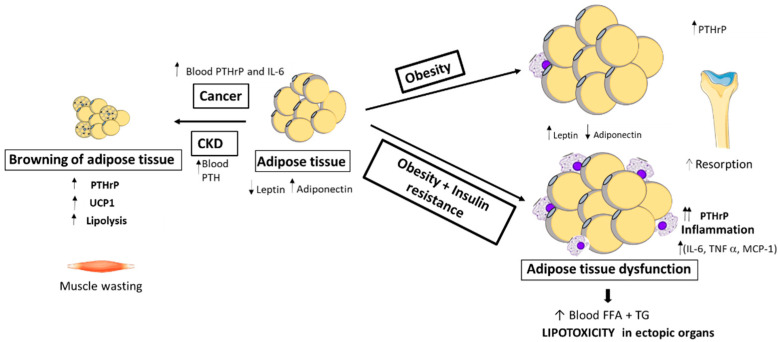
PTHrP and adipose tissue. CKD: Chronic kidney disease; FFA: Free fatty acids; IL-6: Interleukin 6; MCP-1: Monocyte Chemotactic Protein 1; PTHrP: Parathyroid hormone-related protein; TG: Triglycerides; TNFα: Tumor necrosis factor-alpha; UCP-1: Uncoupling protein 1.

PTHrP’s relationship with adipose tissue has recently been established (Figure 2). It has been shown that PTHrP is expressed in human white adipose tissue (WAT), concretely in adipose tissue stromal vascular fraction (SVF)-derived cells from human visceral and subcutaneous WAT [18]. In addition to this, it has been detected that PTH1R is expressed at significant levels in adipose tissue [19].

The expression of PTHrP in the adipose tissue of obese patients with different degrees of insulin resistance was studied and compared with lean patients. The results showed that in visceral adipose tissue, PTHrP positively correlated with the Body Mass Index (BMI) and hip circumference, and PTHrP was expressed to a greater degree in morbidly obese subjects with a high degree of insulin resistance compared with lean and obese subjects with a low degree of insulin resistance [18]. This PTHrP-increased expression associated to obesity and the degree of insulin resistance has also been shown between an *ob*/*ob* mouse, a model morbid obesity, and a POKO mouse, a model of metabolic syndrome [20]. Concretely, the level of PTHrP was greater in the kidney of the POKO mouse, with insulin resistance and more renal lipids when compared to the *ob*/*ob* mouse [20].

On the other hand, in cancer and CKD the role of the PTHrP/PTH1R axis has also been studied, showing that PTH and PTHrP, through the PTH1R receptor, cause the ‘browning’ of white adipose tissue plus energy production via the activation of uncoupling protein-1 (UCP-1) [21,22]. The pathway to browning includes the PTH/PTHrP activation of the protein kinase A (PKA) pathway leading to the expression of thermogenic genes and hypermetabolism of adipose tissues and the loss of muscle mass via the ubiquitin proteasome proteolytic system (UPS), concretely Ucp1, and the induction of muscle atrophy-related genes such as, Muscle RING-finger protein-1 (MuRF-1), Atrogin-1 and Myostatin. In relation to this, it has also been shown that the fat-specific knockout of PTH1R blocks adipose browning and wasting. The loss of PTH1R in fat tissue also preserves muscle mass and improves muscle strength [22]. In addition, He et al. [23], in a primary hyperparathyroidism (PHPT) mouse model that overexpressed PTH, showed adipose browning with an increase of UCP1, elevation of the energy expenditure, decrease in fat content, reduction of body weight, and glucose and insulin tolerance improvement. In this same study [23], it was shown that PHPT patients who also presented high serum PTH levels were associated with a low body weight.

This role of PTHrP as a marker/mediator of stress situations promoting lipolysis and β-oxidation has also been seen in several studies. In mouse adipocytes from epididymal adipose tissue, PTHrP as well as PTH induced lipolysis in a dose-dependent manner. This lipolysis is mediated by protein kinase A (PKA) and also promotes hormone sensitive lipase (HSL) and perilipin phosphorylation [22]. Additionally, in teleost fishes, it has been seen that stanniocalcin 1(STC1) and PTHrP have opposite effects, with STC1 stimulating lipogenesis and PTHrP activating the lipolysis/β-oxidation of fatty acids, suggesting a role for STC1 and PTHrP in strategic energy mechanisms that involve the production of glucose and the safeguarding of liver glycogen reserves for stressful situations [23].

## 4. PTHrP in Obesity, Type 2 Diabetes Mellitus (T2DM) and Metabolic Syndrome (MS)

In conditions of obesity, the excess of energy supply over expenditure is deposited in the WAT in the form of triacylglycerides. WAT either accumulates these lipids inside the adipocytes (hypertrophy) or generates more adipocytes (hyperplasia) until it reaches the limit of expandability [24]. When this limit is surpassed, excess lipids are released into the blood (hyperlipidemia) and begin to accumulate in non-adipose tissue, which contributes to organ damage (such as the pancreas, liver, heart, muscle, kidney, etc.) through a process called lipotoxicity. The lipotoxic process is responsible for the progressive development of insulin resistance (IR), generation of reactive oxygen species (ROS), endoplasmic reticulum stress, alteration of cell signaling pathways, and the release of proinflammatory and profibrotic factors [24]. IR usually precedes pathological situations such as Type 2 Diabetes mellitus (T2DM) or Metabolic Syndrome (MS).

The expression of PTHrP in the adipose tissue of obese patients with different degrees of insulin resistance was studied and compared with lean patients. The results showed that in visceral WAT, PTHrP positively correlated with the Body Mass Index (BMI) and hip circumference, and PTHrP was expressed to a greater degree in morbidly obese subjects with a high degree of insulin resistance compared with lean and obese subjects with a low degree of insulin resistance [18]. This PTHrP-increased expression associated to obesity and the degree of insulin resistance has also been shown between a *ob/ob* mouse, a model of morbid obesity, and a POKO mouse, a model of metabolic syndrome [20]. Concretely, the level of PTHrP was greater in the kidney of the POKO mouse, with insulin resistance and more renal lipids when compared to the *ob/ob* mouse [20]. These studies suggest that PTHrP could be used as a marker of worsening or metabolic dysfunction associated with obesity. However, Qin et al. [25] have recently shown in a high fat diet (HFD)-induced murine obesity model that PTHrP overexpression promotes WAT browning and inhibits BAT whitening, preventing obesity, hepatic steatosis and insulin resistance [25], thereby proposing the application of PTHrP to avoid metabolic complications in obese patients.

On the other hand, it is known that PTHrP and PTH1R are expressed in the pancreas, and it has been seen that PTHrP increases insulin secretion and the proliferation and survival of the beta cells of pancreatic islets [26]. In T2DM patients, there is an increase in the serum concentration of PTHrP, and despite presenting a normal bone mass they have defects in the structure and strength of the bone. Recently, it was shown that Abaloparatide (a synthetic analog of human PTHrP) treatment of postmenopausal women with T2DM resulted in significant improvements in Bone Mineral Density (BMD), improving the bone microarchitecture and increasing the bone strength [27]. Many T2DM patients develop diabetic nephropathy, with renal overexpression of both PTHrP and PTH1R, associated with the development of renal hypertrophy and proteinuria [28,29], correlating positively with the level of hyperglycemia [29].

## 5. PTHrP, Adipose Tissue and Pregnancy

During pregnancy, adaptations of the maternal physiology occur to guarantee the supply of nutrients and oxygen, favoring the correct fetal development. The women are subjected to hormonal variations; there is a progressive increase in weight, in the fat mass and in its distribution. In addition to this, a slight general inflammatory state and modifications in metabolism appear [30].

PTHrP is expressed in the uterus, placenta, fetal membranes and the fetus itself. Its function is to regulate the transport of calcium through the placenta, and it favors the vasodilation of the maternal-fetal vessels. In addition, PTHrP produces the relaxation of the smooth muscles of the uterus and stimulates cell growth and differentiation. In humans, the concentration of PTHrP in the blood increases throughout pregnancy, and its levels correlate with the calcium concentration [31,32].

**Figure 3:**
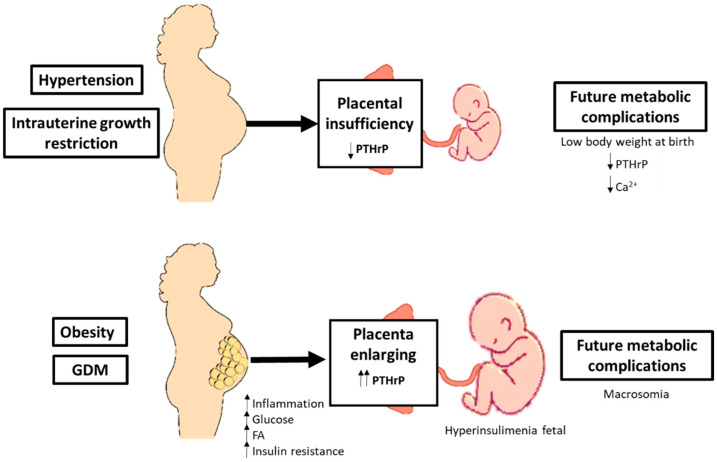
PTHrP, adipose tissue and pregnancy. FA: Fatty acids; GDM: Gestational diabetes mellitus; PTHrP: Parathyroid hormone-related protein.

In intrauterine growth restriction animal models, one has seen a decreased content of PTHrP [33,34]. In uteroplacental insufficiency, which mimics placental insufficiency in humans, one has seen a decreased content of PTHrP in the placenta and in the calcium perinatal, associated with a fetal growth restriction and reduced litter size of viable fetuses [34]. Furthermore, in spontaneously hypertensive rats (SHR), one has shown decreased PTHrP concentrations in the fetal plasma, placenta and amniotic fluid, and in the late gestation, the intrauterine treatment with an antagonist PTH1R receptor increased fetal and placental weights, associated with a fetal and maternal PTHrP upregulation in the placenta, uterus and plasma [35] (Figure 3).

However, when there is overweight or obesity in pregnancy, it produces an increase in PTHrP in the placenta [36]. Maternal overweight is often linked to the development of gestational diabetes mellitus (GDM). GDM and maternal obesity share metabolic abnormalities (such as hyperinsulinemia, severe insulin resistance, and chronic low-grade inflammation) and are related to the development of future metabolic alterations, such as obesity or T2DM, both in mothers and offspring [37]. In this sense, it has been seen that the expression of PTHrP and PTH1R in the placenta depends on maternal hyperglycemia and obesity and is directly related to adverse pregnancy outcomes [36].

## 6. Conclusions

The PTHrP/PTH1R axis plays a central role in adipose tissue, participating in its differentiation and remodeling. On the one hand, the implications of this axis in the process of differentiation of stem cells towards adipogenesis or osteogenesis have been seen. On the other hand, it seems that PTHrP is key in situations of “stress” of adipose tissue, both by excess (in obesity, metabolic syndrome, T2DM and GDM) and by default (cancer and CKD) in the disease. Because of this, PTHrP might be used in obesity treatment for its ability to reprogram adipogenesis and adipose tissue expansion, favoring lipolysis, WAT browning and the improvement of the insulin sensitivity. However, it could also be used on patients with cachexia due to long-lasting diseases or with wear and tear of the body, such as cancer or CKD, and to improve the treatments of bone diseases such as osteoporosis. Finally, PTHrP could even be used as a marker of placental status and maternal adaptations to prevent future metabolic problems in mothers and children.

## Data Availability

Not report any data.

## Abbreviations

Adrp: Adipose differentiation-related protein: BAT: Brown adipose tissue; BMD: Bone Mineral Density; C/EBP: CCAAT/enhancer binding protein; CKD: Chronic kidney disease; FFA: Free fatty acids; GDM: Gestational Diabetes mellitus; HFD: High Fat Diet; HHM: Humoral hypercalcemia of malignancy; HSL: Hormone sensitive lipase; IL-6: Interleukin 6; IR: Insulin resistance; LCPUFAs: long chain polyunsaturated fatty acids; MCP-1: Monocyte Chemotactic Protein 1; MS: Metabolic Syndrome; MSCs: mesenchymal stem cells; MuRF-1: Muscle RING-finger protein-1; PHPT: Primary Hyperparathyroidism; PKA: Protein kinase A; PPARγ: Peroxisome proliferator-activated receptor gamma; PTHrP: Parathyroid hormone-related protein; RBP-4: Retinol-binding protein 4; ROS: Reactive oxygen species; RunX2: Runt-related transcription factor 2; SHR: Spontaneously hypertensive rats; STC1: Stanniocalcin 1; SVF: Stromal vascular fraction; TG: Triglycerides; TNFα: Tumor necrosis factor-alpha; T2DM: Type 2 Diabetes mellitus; UCP-1: Uncoupling protein 1; UPS: Ubiquitin proteasome proteolytic system; WAT: White adipose tissue.

## References

[1] Massfelder T., Helwig J.-J. (2003). The Parathyroid Hormone-Related Protein System: More Data but More Unsolved Questions. Curr. Opin. Nephrol. Hypertens..

[2] Usdin T.B., Gruber C., Bonner T.I. (1995). Identification and Functional Expression of a Receptor Selectively Recognizing Parathyroid Hormone, the PTH2 Receptor. J. Biol. Chem..

[3] Usdin T.B., Hoare S.R.J., Wang T., Mezey É., Kowalak J.A. (1999). TIP39: A new neuropeptide and PTH2R agonist from hypothalamus. Nat. Neurosci..

[4] Cinti S. (2012). The Adipose Organ at a Glance. DMM Dis. Models Mech..

[5] Pellegrinelli V., Carobbio S., Vidal-Puig A. (2016). Adipose Tissue Plasticity: How Fat Depots Respond Differently to Pathophysiological Cues. Diabetologia.

[6] Chen Q., Shou P., Zheng C., Jiang M., Cao G., Yang Q., Cao J., Xie N., Velletri T., Zhang X. (2016). Fate Decision of Mesenchymal Stem Cells: Adipocytes or Osteoblasts?. Cell Death Differ..

[7] Chan G.K., Deckelbaum R.A., Bolivar I., Goltzman D., Karaplis A.C. (2001). PTHrP Inhibits Adipocyte Differentiation by Down-Regulating PPARγ Activity via a MAPK-Dependent Pathway. Endocrinology.

[8] Chan G.K., Miao D., Deckelbaum R., Bolivar I., Karaplis A., Goltzman D. (2003). Parathyroid Hormone-Related Peptide Interacts with Bone Morphogenetic Protein 2 to Increase Osteoblastogenesis and Decrease Adipogenesis in Pluripotent C3H10T1/2 Mesenchymal Cells. Endocrinology.

[9] Amizuka N., Karaplis A.C., Henderson J.E., Warshawsky H., Lipman M.L., Matsuki Y., Ejiri S., Tanaka M., Izumi N., Ozawa H. (1996). Haploinsufficiency of Parathyroid Hormone-Related Peptide (PTHrP) Results in Abnormal Postnatal Bone Development. Dev. Biol..

[10] Casado-Díaz A., Santiago-Mora R., Quesada J.M. (2010). The N- and C-Terminal Domains of Parathyroid Hormone-Related Protein Affect Differently the Osteogenic and Adipogenic Potential of Human Mesenchymal Stem Cells. Exp. Mol. Med..

[11] Xiong Z., Luo P., Zhou J., Tan M. (2019). 15-Deoxy-Δ12,14-Prostaglandin J2 as a Potential Regulator of Bone Metabolism via Pparγ- Dependent and Independent Pathways: A Review. Drug Des. Dev. Ther..

[12] Medina-Gomez G., Gray S., Vidal-Puig A. (2007). Adipogenesis and Lipotoxicity: Role of Peroxisome Proliferator-Activated Receptor γ (PPARγ) and PPARγcoactivator-1 (PGC1). Public Health Nutr..

[13] Rosen E.D., Sarraf P., Troy A.E., Bradwin G., Moore K., Milstone D.S., Spiegelman B.M., Mortensen R.M. (1999). PPAR gamma is required for the differentiation of adipose tissue in vivo and in vitro. Mol. Cell..

[14] Kim J.H., Yamaori S., Tanabe T., Takagi M., Matsubara T., Okamoto M., Kimura S., Gonzalez F.J. (2017). Lack of Epithelial PPARγ Causes Cystic Adenomatoid Malformations in Mouse Fetal Lung. Biochem. Biophys. Res. Commun..

[15] Rehan V.K., Torday J.S. (2012). PPARγ Signaling Mediates the Evolution, Development, Homeostasis, and Repair of the Lung. PPAR Res..

[16] Candelario J., Tavakoli H., Chachisvilis M. (2012). PTH1 Receptor Is Involved in Mediating Cellular Response to Long-Chain Polyunsaturated Fatty Acids. PLoS ONE.

[17] Choe S.S., Huh J.Y., Hwang I.J., Kim J.I., Kim J.B. (2016). Adipose Tissue Remodeling: Its Role in Energy Metabolism and Metabolic Disorders. Front. Endocrinol. (Lausanne).

[18] Roca-Rodríguez M.M., El Bekay R., Garrido-Sanchez L., Gómez-Serrano M., Coin-Aragüez L., Oliva-Olivera W., Lhamyani S., Clemente-Postigo M., García-Santos E., de Luna Diaz R. (2015). Parathyroid Hormone-Related Protein, Human Adipose-Derived Stem Cells Adipogenic Capacity and Healthy Obesity. J. Clin. Endocrinol. Metab..

[19] Larsson S., Jones H.A., Göransson O., Degerman E., Holm C. (2016). Parathyroid Hormone Induces Adipocyte Lipolysis via PKA-Mediated Phosphorylation of Hormone-Sensitive Lipase. Cell. Signal..

[20] Martínez-García C., Izquierdo A., Velagapudi V., Vivas Y., Velasco I., Campbell M., Burling K., Cava F., Ros M., Orešič M. (2012). Accelerated Renal Disease Is Associated with the Development of Metabolic Syndrome in a Glucolipotoxic Mouse Model. DMM Dis. Models Mech..

[21] Kir S., White J.P., Kleiner S., Kazak L., Cohen P., Baracos V.E., Spiegelman B.M. (2014). Tumour-Derived PTH-Related Protein Triggers Adipose Tissue Browning and Cancer Cachexia. Nature.

[22] Kir S., Komaba H., Garcia A.P., Economopoulos K.P., Liu W., Lanske B., Hodin R.A., Spiegelman B.M. (2016). PTH/PTHrP Receptor Mediates Cachexia in Models of Kidney Failure and Cancer. Cell Metab..

[23] Palma P.F.S., Bock C., Silva T.S., Guerreiro P.M., Power D.M., Pörtner H.O., Canário A.V.M. (2019). STC1 and PTHrP Modify Carbohydrate and Lipid Metabolism in Liver of a Teleost Fish. Sci. Rep..

[24] Virtue S., Vidal-Puig A. (2010). Adipose Tissue Expandability, Lipotoxicity and the Metabolic Syndrome—An Allostatic Perspective. Biochim. Et Biophys. Acta.

[25] Qin B., Qincao L., He S., Liao Y., Shi J., Xie F., Diao N., Bai L. (2021). Parathyroid Hormone-Related Protein Prevents High-Fat-Diet-Induced Obesity, Hepatic Steatosis and Insulin Resistance in Mice. Endocr. J..

[26] Mozar A., Lin H., Williams K., Chin C., Li R., Kondegowda N.G., Stewart A.F., Garcia-Ocaña A., Vasavada R.C. (2016). Parathyroid Hormone-Related Peptide (1-36) Enhances Beta Cell Regeneration and Increases Beta Cell Mass in a Mouse Model of Partial Pancreatectomy. PLoS ONE.

[27] Miller P.D., Bilezikian J.P., Fitzpatrick L.A., Mitlak B., McCloskey E.V., Cosman F., Bone H.G. (2020). Abaloparatide: An Anabolic Treatment to Reduce Fracture Risk in Postmenopausal Women with Osteoporosis. Curr. Med. Res. Opin..

[28] Romero M., Ortega A., Olea N., Arenas M.I., Izquierdo A., Bover J., Esbrit P., Bosch R.J. (2013). Novel Role of Parathyroid Hormone-Related Protein in the Pathophysiology of the Diabetic Kidney: Evidence from Experimental and Human Diabetic Nephropathy. J. Diabetes Res..

[29] Izquierdo A., López-Luna P., Ortega A., Romero M., Gutiérrez-Tarrés M.A., Arribas I., Álvarez M.J.R., Esbrit P., Bosch R.J. (2006). The Parathyroid Hormone-Related Protein System and Diabetic Nephropathy Outcome in Streptozotocin-Induced Diabetes. Kidney Int..

[30] Corrales P., Vidal-Puig A., Medina-Gómez G. (2018). PPARS and Metabolic Disorders Associated with Challenged Adipose Tissue Plasticity. Int. J. Mol. Sci..

[31] Napso T., Yong H.E.J., Lopez-Tello J., Sferruzzi-Perri A.N. (2018). The Role of Placental Hormones in Mediating Maternal Adaptations to Support Pregnancy and Lactation. Front. Physiol..

[32] Hysaj O., Marqués-Gallego P., Richard A., Elgizouli M., Nieters A., Quack Lötscher K.C., Rohrmann S. (2021). Parathyroid Hormone in Pregnancy: Vitamin D and Other Determinants. Nutrients.

[33] Wlodek M.E., Westcott K.T., O′dowd R., Serruto A., Wassef L., Moritz K.M., Moseley J.M. (2005). Uteroplacental Restriction in the Rat Impairs Fetal Growth in Association with Alterations in Placental Growth Factors Including PTHrP. Am. J. Physiol. Regul. Integr. Comp. Physiol..

[34] Wlodek M.E., Westcott K.T., Ho P.W., Serruto A., Di Nicolantonio R., Farrugia W., Moseley J.M. (2000). Reduced fetal, placental, and amniotic fluid PTHrP in the growth-restricted spontaneously hypertensive rat. Am. J. Physiol. Regul. Integr. Comp. Physiol..

[35] Woldek M.E., di Nicolantonio R., Westcott K.T., Farrugia W., Ho P.W.M., Moseley J.M. (2004). PTH/PTHrP Receptor and Mid-Molecule PTHrP Regulation of Intrauterine PTHrP: PTH/PTHrp Receptor Antagonism Increases SHR Fetal Weight. Placenta.

[36] Sirico A., Dell’aquila M., Tartaglione L., Moresi S., Farì G., Pitocco D., Arena V., Lanzone A. (2021). PTH-RP and PTH-R1 Expression in Placentas from Pregnancies Complicated by Gestational Diabetes: New Insights into the Pathophysiology of Hyperglycemia in Pregnancy. Diagnostics.

[37] Mamun A.A., Mannan M., Doi S.A.R. (2014). Gestational Weight Gain in Relation to Offspring Obesity over the Life Course: A Systematic Review and Bias-Adjusted Meta-Analysis. Obes. Rev..

